# Drug Repurposing, a Fast-Track Approach to Develop Effective Treatments for Glioblastoma

**DOI:** 10.3390/cancers14153705

**Published:** 2022-07-29

**Authors:** Ioannis Ntafoulis, Stijn L. W. Koolen, Sieger Leenstra, Martine L. M. Lamfers

**Affiliations:** 1 Brain Tumor Center, Department of Neurosurgery, Erasmus MC Cancer Institute, Erasmus University Medical Center, 3015 CN Rotterdam, The Netherlands; i.ntafoulis@erasmusmc.nl (I.N.); s.leenstra@erasmusmc.nl (S.L.); 2 Department of Medical Oncology, Erasmus MC Cancer Institute, Erasmus University Medical Center, 3015 CN Rotterdam, The Netherlands; s.koolen@erasmusmc.nl; 3 Department of Hospital Pharmacy, Erasmus University Medical Center, 3015 CN Rotterdam, The Netherlands

**Keywords:** drug repurposing, glioblastoma, blood–brain barrier, efflux pumps, drug screening platforms, CNS penetration, clinical trials

## Abstract

**Simple Summary:**

Introducing novel and effective treatments against glioblastoma (GBM) remains an arduous journey as reflected in the negative outcome of most clinical trials. The blood–brain barrier and the tremendous heterogeneity of the disease comprise major obstacles in this process. Drug repurposing is a drug discovery approach that can accelerate the drug development timeline and identify promising candidates for GBM treatment. Obtaining insights already at preclinical stage into drug sensitivity and physicochemical properties for central nervous system (CNS) penetration of these candidates could shift research outcomes to more effective drugs for clinical investigation against GBM.

**Abstract:**

Glioblastoma (GBM) remains one of the most difficult tumors to treat. The mean overall survival rate of 15 months and the 5-year survival rate of 5% have not significantly changed for almost 2 decades. Despite progress in understanding the pathophysiology of the disease, no new effective treatments to combine with radiation therapy after surgical tumor debulking have become available since the introduction of temozolomide in 1999. One of the main reasons for this is the scarcity of compounds that cross the blood–brain barrier (BBB) and reach the brain tumor tissue in therapeutically effective concentrations. In this review, we focus on the role of the BBB and its importance in developing brain tumor treatments. Moreover, we discuss drug repurposing, a drug discovery approach to identify potential effective candidates with optimal pharmacokinetic profiles for central nervous system (CNS) penetration and that allows rapid implementation in clinical trials. Additionally, we provide an overview of repurposed candidate drug currently being investigated in GBM at the preclinical and clinical levels. Finally, we highlight the importance of phase 0 trials to confirm tumor drug exposure and we discuss emerging drug delivery technologies as an alternative route to maximize therapeutic efficacy of repurposed candidate drug.

## 1. Introduction

Glioblastoma (GBM) is the most aggressive form of diffuse gliomas and the most lethal among all types of brain tumors, comprising 12–15% of all adult intracranial tumors and 50–60% of astrocytic neoplasms [1]. According to the 2021 WHO classification of CNS tumors, former (grade 4) GBM is now classified based on the presence or absence of mutations in the isocitrate dehydrogenase (IDH) gene: IDH wild-type (IDHwt) glioblastoma or IDH-mutant (IDHmut) grade 4 astrocytoma [2]. Molecularly, IDHwt glioblastomas are characterized by the presence of TERT promoter mutation, EGRF amplification, +7/−10 chromosome copy number changes or any combination of the above [2]. IDHmut grade 4 astrocytomas are characterized by mutations of IDH1/2, ATRX, TP53, CDKN2A/B homozygous deletion, PDGFRA amplification or any combination of the above [2]. These genomic alterations of IDHwt gliomas are associated with fast growth rates and poor prognosis [2].

The standard of care treatment following diagnosis comprises maximal safe surgical resection of the tumor (debulking), followed by radiation therapy (RT) and concurrent and adjuvant chemotherapy with the alkylating agent temozolomide (TMZ) [3,4]. However, the survival rates of the patients diagnosed with GBM and receiving first-line treatment remain very low. The median overall survival (OS) is 12–15 months, while only 3% of patients have a progression-free survival (PFS) of more than 5 years [5]. The MGMT (O6-methylguanine–DNA methyltransferase) promoter is a well-established predictive marker of response in GBM patients receiving TMZ. The epigenetic silencing of the MGMT gene by promoter methylation compromises DNA repair, improving response to TMZ and leading to longer survival of glioblastoma patients [6]. Inevitably, all GBM patients receiving RT + TMZ and/or adjuvant TMZ therapy relapse; the median PFS upon completing the first line of treatment varies between 1, 5 and 6 months [7]. Lomustine (CCNU), an alkylating agent, is sometimes administered as ultimate treatment option to recurrent GBM patients with minor therapeutic benefit [8,9,10,11].

In 2009, the U.S. food and drug administration (FDA) approved bevacizumab for the treatment of GBM with relapse after prior RT + chemotherapy [12]. Bevacizumab is a recombinant humanized monoclonal antibody, with anti-angiogenic properties by blocking vascular endothelial growth factor A (VEGF-A). However, its moderate clinical benefit and unproven OS advantage to date have withheld approval by the European medicine agency (EMA) [13,14,15,16]. The most recent therapeutic approach for recurrent GBM, which received FDA approval in 2011, is a device known as tumor-treating fields (TTF) [17]. In 2015, the device was also granted FDA approval for newly diagnosed GBM [17]. The device delivers low intensity, alternating electric fields to the tumor, therewith inhibiting glioma cell proliferation [18,19]. Moderate improvements in the survival of newly diagnosed GBM patients have been observed by adding TTF as an adjuvant treatment upon completing the standard of care treatment [20,21]. In Europe, the use of TTF is very limited to date, as the appropriate usage and implementation of the device in daily clinical practice presents many challenges [22,23].

Despite these limited additions to the arsenal of treatments, the prognosis of GBM patients remains dismal [24,25]. Two key players are involved in failure of conventional and targeted therapies: (1) the tremendous intra- and inter-tumoral heterogeneity of GBM and (2) the blood–brain barrier (BBB) [26,27,28]. GBM heterogeneity contributes to drug resistance and treatment escape and comprises a complex and arduous obstacle to overcome [29,30,31]. Extensive genetic and epigenetic profiling led to the classification of GBM tumors into three distinct molecular subgroups (classical, mesenchymal and proneural) as well as to the characterization of distinct DNA methylation profiles and/or expression patterns within these GBM subgroups [32,33,34]. Additionally, single-cell RNA sequencing analysis revealed different molecular subtypes within each tumor that can dynamically adapt to micro-environmental cues [34,35,36,37]. To date, these findings provide a better understanding of the heterogeneous nature of GBM; however, their clinical relevance, in particular in relation to drug treatment, is still limited [38].

The second major obstacle in GBM treatment is the BBB, which prevents effective delivery of drugs to the central nervous system (CNS). Therefore, to achieve any therapeutic response, it is of utmost importance that drugs cross the BBB and reach the tumor region in therapeutically effective concentrations. Drug discovery tools have been developed to identify optimal drug candidates for CNS penetration based on their physicochemical properties [39,40]. Moreover, efforts are being directed towards assessing CNS penetration and actual target delivery of new agents, as noted in the increasing number of phase 0 trials for GBM [41,42,43]. In addition, new delivery techniques, such as focused ultrasound sonication (FUS) and/or the use of nanoparticles to encapsulate therapeutic molecules, are being used to enhance systemic drug delivery into the CNS [44,45,46]. Examples include chemotherapeutic agents widely used in clinical practice, such as paclitaxel, cytarabine, carboplatin, etoposide and daunorubicin [47,48,49,50,51,52,53]. 

Based on these developments, a renewed interest in the available anticancer agents has arisen. With tools available to predict, enhance and assess drug delivery to CNS tumors as well as approaches to define markers of tumor sensitivity to specific compounds, the available arsenal of approved anticancer agents may be re-evaluated for potential GBM treatment. This approach, known as drug repurposing or drug repositioning, is a recognized strategy in drug discovery aiming to identify secondary indications for already approved drugs [54,55,56]. Given the unmet need for novel therapeutic options for GBM, drug repurposing may be a valuable tool, bypassing the delays and high costs of the novel drug development process and providing new drug candidates against GBM within a relatively short timeframe.

In this review, we aim to: (1) describe the role of the BBB and tumor heterogeneity in the failure of treatments; (2) introduce the significance of drug repurposing in identifying new candidate agents against GBM; (3) highlight the importance of selecting candidates based on the physicochemical properties and/or PK profiles for CNS penetration, as well as the development of novel delivery approaches, to optimize drug delivery to GBM; and (4) provide recent examples of repurposed drugs under clinical investigation against GBM.

## 2. The Blood–Brain Barrier and Drug Efflux Pumps

**Figure 1:**
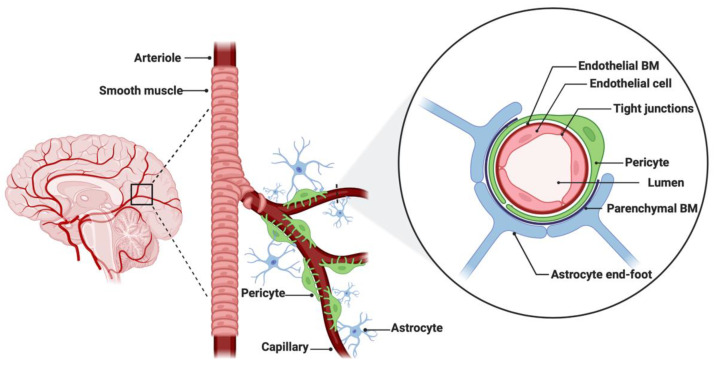
Anatomical features of the blood–brain barrier (BBB). The structure of the BBB in normal physiology consists of endothelial cells interconnected with a complex network of proteins (tight junctions), while mechanical support is provided by pericytes and astrocytic end-feet. The parenchymal and endothelial basal membranes (BM) provide additional strengthening to the cell attachments. Figure was created in BioRender.com (accessed on 5 July 2022).

**Figure 2:**
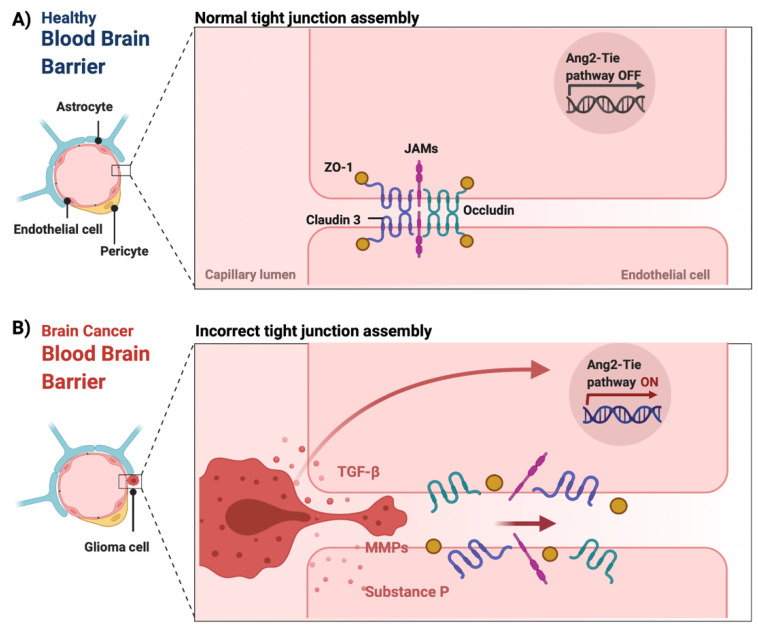
The integrity of the blood–brain barrier in physiological and malignant conditions. (**A**) In physiological conditions, tight junctions (claudin 3, occludin, junction adhesion molecules (JAMS) as well as cytoplasmic accessory proteins, such as Zonula occludens-1 (ZO-1)) of the endothelial cells remain intact maintaining the integrity of the BBB. (**B**) In CNS tumors, the release of chemical mediators by the tumor cells, such as substance P, matrix metalloproteinases (MMPs) and transforming growth factor beta (TGF-β), can cause the loss of tight junctions, which leads to the dysfunction and disruption of the BBB [60,69,70,72]. Additionally, the overexpression of angiopoietin-2 (ANG-2) is linked to vascular malformations and pericyte detachment through the hypoxic upregulation of VEGF, which subsequently promotes angiogenesis at the tumor margin [68]. Figure was created in BioRender.com (accessed on 20 May 2022).

The BBB is a neurovascular unit that, in physiological conditions, acts as a ‘gatekeeper’ [57]. The main task of the BBB is to maintain the brain homeostasis by controlling the passage of endogenous and exogenous molecules from the blood stream into the CNS [57,58]. Structurally, the BBB consists of endothelial cells interconnected with a complex network of proteins (tight junctions), while pericytes and astrocytic end-feet provide an additional structural support to the brain microvasculature (Figure 1) [57,59]. Tight junctions (TJs) are the key feature of the BBB and responsible for the impediment of polar solutes through intracellular and paracellular diffusion pathways [57]. TJs consist of claudins, occludins, junction adhesion molecules (JAMS) and various cytoplasmic accessory proteins, such as Zonula occludens-1, -2, -3 (ZO-1, ZO-2, ZO-3) (Figure 2) [60]. The transport of molecules across the BBB can be achieved by different routes, including passive diffusion, solute carriers (SLC), ATP-binding cassette (ABC) transporters, transcytosis and receptor-mediated transport [61,62]. Lipid soluble molecules can passively diffuse the BBB and reach the CNS at a rate that is linked to their physicochemical properties [40].

In pathological conditions, such as brain tumors, the BBB is presented with functional abnormalities affecting normal cellular processes. [63]. Such functional abnormalities also affect processes such as angiogenesis, leading to an abnormal production of proangiogenic factors and malformation of blood vessels [64,65,66]. Specifically, the activation of the endothelial angiopoietin-2 (ANG-2)-TIE growth factor receptor pathway promotes the upregulation of VEGF and the induction of tumor angiogenesis [64,67,68]. Additionally, imbalances in the release of chemical mediators, such as substance P, histamine, bradykinin, thrombin matrix metalloproteinases (MMPs) and/or cytokines, including tumor necrosis factor-alpha (TNF-α), transforming growth factor beta (TGF-β), interleukin (IL)-1 beta and IL-6, can cause the loss of TJs and subsequently BBB breakdown and dysfunction (Figure 2) [57,60,69,70,71,72]. The loss of aquaporin 4 (AQP4) can also lead to BBB disruption by inducing the polarization of astrocytic end-feet [73]. These changes can result in a leaky BBB, also known as the blood–tumor barrier (BTB) [74]. In fact, this leakiness forms the basis of contrast-enhanced MRI imaging of CNS tumors. The extent to which the accumulation of therapeutic agents into brain tumor tissue is affected by the BTB is not well known.

Glioblastoma displays intra-tumoral heterogeneity in drug penetration, resulting from localized areas of vasogenic edema and areas with an intact BBB [74]. The highly infiltrative nature of glioma cells allows them to invade the surrounding brain parenchyma, therewith instigating the growth of malignant foci at a distance of the tumor core around blood vessels with an intact BBB [75,76,77]. After the surgical resection of the tumor core, these cells are left behind and are responsible for the recurrence of the tumor. It, therefore, remains crucial for the development of new treatments that drugs effectively penetrate the BBB in order to reach these foci.

Another key player impeding drug delivery into the CNS is the family of drug efflux pumps and more specifically the ATP-binding cassette (ABC) transporters [78]. The family of ABC transporters consists of 48 identified human ABC transporter genes classified in seven subfamilies [79]. The ABC transporters are actively involved in many intracellular processes by importing or exporting substrates through membranes by utilizing ATP [78,79,80]. Mutations in genes encoding ABC transporters can lead to numerous disorders comprising retinal degeneration, skin diseases, cystic fibrosis and hypercholesterolemia [81,82]. In human malignancies, the role of ABC transporters in the development of multidrug resistance (MDR) has been extensively studied [83,84,85,86,87]. The main efflux pumps linked to MDR are the (1) ABCB1 (P-glycoprotein or P-gp), (2) ABCG2 (breast cancer resistance protein, (BCRP)) and (3) ABCC4 (multidrug resistance-associated protein 4 (MRP4)) [88,89]. Approximately 60% of the available drugs on the market are substrates of ABCB1, making it a key player in the regulation of intracellular drug accumulation and cytotoxicity [90].

Under physiological conditions, ABCB1 and ABCG2 are mainly expressed by brain endothelial cells, allowing the efflux of molecules from the brain parenchyma to the bloodstream [91,92]. In brain tumors such as glioma, efflux pumps are present on the (peri)tumoral vasculature as well as on glioma cells [93]. The upregulation of ABCB1 and ABCG2 hampers the CNS delivery of chemotherapeutic agents, including TMZ [92,94,95,96]. Additionally, de Gooijer et al. have shown that drug delivery restriction is observed even when the BBB is disrupted, highlighting the key role of tumor-cell-associated efflux pumps in the development of drug resistance against GBM [78]. The unique anatomical and biological features of GBM make its treatment extremely challenging. Undoubtedly, the role of drug efflux transporters can be linked to the innumerable failures of clinical trials in GBM and, therefore, needs to be taken into consideration in order to design more effective treatments [91,97]. Hence, in drug development, it is a pre-requisite to identify or design drugs with optimal physicochemical properties and PK profiles to cross the BBB, but also with a low affinity for the ABC transporters in order to achieve and maintain therapeutically effective concentrations in brain tumor tissue [75,98,99,100].

## 3. Tumor Heterogeneity and Drug Resistance

Tumor heterogeneity is another important factor involved in the development of drug resistance, which subsequently leads to limitations or failures of the majority of therapeutic approaches against GBM [26,28]. It can be classified as intra- and inter-tumoral heterogeneity. Intra-tumoral heterogeneity allows molecularly distinct subpopulations to escape treatment, signifying the need for combination therapies to target the whole tumor population [101,102]. On the other hand, the large inter-tumoral heterogeneity, characterized by intrinsically resistant or sensitive tumors to any given drug, reveals the need to identify biomarkers of response and to focus on personalized treatments for GBM. Indeed, drug screening studies on panels of patient-derived GBM cells have uncovered tremendous intertumoral variability in drug sensitivities for almost any given drug [103,104,105]. The most well-studied biomarker in GBM is the methylation status of the MGMT promoter, which is predictive for response to temozolomide [106,107]. Nowadays, great efforts are being made to identify the biomarkers of response for a multitude of potential GBM treatments, including targeted drugs, chemotherapies and immunotherapies [105,108,109,110]. Recently, Fabro et al. described the different biological mechanisms that glioma cells exploit to escape TMZ treatment, which include enhanced DNA repair, epigenetic changes, stem cell characteristics, the tumor microenvironment, metabolism, autophagy, adaptive molecular pathways and enhanced drug efflux [29]. A better understanding of these mechanisms can aid in identifying the biomarkers of response as well as in the development of combination therapies to counteract drug resistance mechanisms [26,27]. 

The majority of the clinical trials in GBM patients during the past two decades yielded disappointing outcomes, at best prolonging survival in small sub-populations of patients [111]. These failures have been attributed to, among other factors, the lack of BBB penetrance of tested drugs as well as rapidly developing drug resistance, but also to the interpatient variability in intrinsic tumor sensitivity for the tested agent [112]. Many of these trials were considered a failure when only a small percentage (~10%) of patients responded to the treatment. With more emphasis on the selection of compounds with favorable physicochemical properties, as well as the availability of predictive biomarkers of response, some of those drugs may be of interest to be re-evaluated.

## 4. Drug Repurposing for Glioblastoma

**Figure 3:**
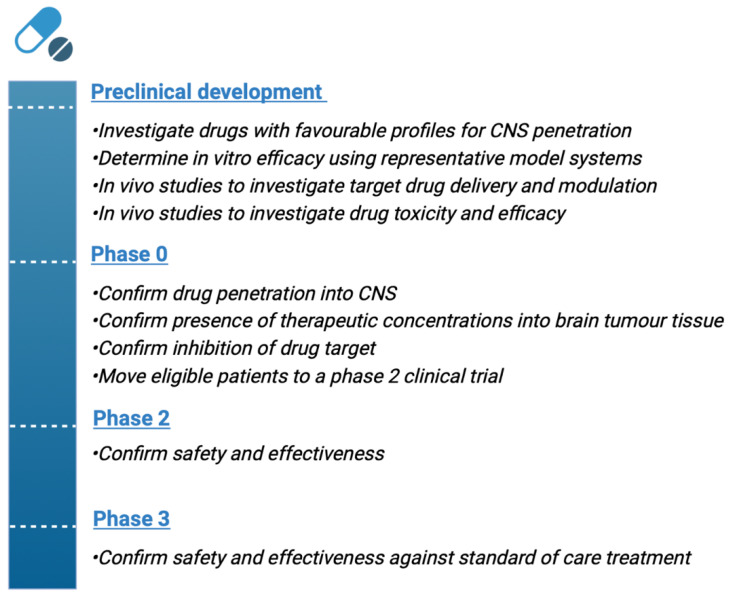
Development stages for repurposing a drug in glioblastoma.

Drug repurposing is a drug discovery approach investigating new indications for registered medicines against various diseases, including cancer [113]. Given the failures of introducing new and effective treatments for GBM over the past decades [114], drug repurposing might offer a way to identify drug candidates with favorable characteristics for CNS penetration that can be provided to patient-(sub)populations in a relatively short timeframe. A significant advantage of this approach is the available knowledge of pharmacokinetics (PK), pharmacodynamics (PD) and drug safety profiles. This knowledge can lead to the considerable shortening of the preclinical and clinical phases of drug development (Figure 3) [115].

Drug repurposing has already been applied in different types of cancers. Examples of successfully repurposed drugs that were granted approval for a second indication include: aspirin for colorectal cancer [116,117], raloxifene for breast cancer [118,119] and thalidomide for multiple myeloma [55,120]. Additional examples of drug repurposing applications in clinical cancer drug development are the MyPathway and DRUP studies [121,122]. The most recent one, the DRUP study, it is a Dutch multicenter personalized therapy trial that aims to expand the use of available targeted anticancer drugs to patients with other types of cancer but who share the same genetic profile [121]. Unfortunately, only a small percentage of GBM patients are enrolled in such trials as genomic-based approaches rarely identify targetable mutations in this patient group for which effective compounds are available. One targetable mutation that can be identified in GBM, and for which a therapeutically effective compound is available, is the BRAF-V600 mutation [123,124]. Recently, a multicenter, open-label, single-arm, phase 2, basket trial showed that the combination of dabrafenib (BRAF inhibitor) with trametinib (MEK inhibitor) has clinical efficacy against low- and high-grade gliomas [125]. Both drugs have previously been granted FDA approval as a combination for the treatment of metastatic BRAF-V600 mutated melanoma [126,127].

Additionally, the DNA damage repair protein MGMT plays a major role in the development of drug resistance to alkylating agents by removing alkyl groups from the O6 position of guanine [128,129]. Disulfiram (ALDH inhibitor) was found to inhibit MGMT activity and sensitize glioma cells to alkylating agents [130]. Disulfiram is an FDA approved drug used for alcoholism treatment [131]. Recently, Wu et al. have shown that the inhibition of PARylation by talazoparib (a PARP inhibitor) reduces MGMT functioning and therefore increases the sensitivity to TMZ of GBM cells with unmethylated MGMT promoter. Talazoparib is an FDA/EMA approved agent for the treatment of advanced breast cancer with BRCA mutation [132].

### 4.1. Drug Screening Platforms for the (Re)Evaluation of Registered Compounds

Drug repurposing has also found its way to preclinical drug development research. Patient-derived preclinical model systems (2D and 3D cell cultures and PDX models) have become the gold-standard for GBM drug discovery and development [31,133,134,135,136,137,138,139,140]. These preclinical models have been shown to recapitulate the human GBM biology and to predict clinical response to the standard of care agent temozolomide in GBM patients [141,142,143]. Large-scale drug screening efforts applying such platforms can identify drug candidates for GBM based on dose–response profiles. Importantly, panels of patient-derived GBM models representing the whole spectrum of GBM patients can be employed, allowing for the identification of biomarkers of response to tested agents as well as pathways of resistance. Such approaches can also aid in the identification of effective combination strategies [104,144].

Promising drug candidates identified based on such approaches include actinomycin-D. Taylor et al. identified actinomycin-D as a promising candidate among the approved oncology drug set VIII against recurrent GBM, showing that actinomycin-D is more effective than TMZ [145]. Recently, our group also identified actinomycin-D as a highly active compound in IDHmut high-grade glioma cell cultures [146]. Actinomycin-D is an antineoplastic antibiotic agent, approved in the 1960s and is still widely used for the treatment of various human malignancies [147]. Mechanistically, actinomycin-D exhibits anticancer activity by targeting RNA polymerase and inhibiting transcription [147]. Clinical trials testing actinomycin-D in GBM patients have not yet been reported, which may be related to its poor PK profile for CNS penetration. However, given the emerging new delivery approaches such as FUS and liposomal delivery forms, actinomycin-D may comprise a promising repurposed drug candidate for further investigation.

Interestingly, omacetaxine mepesuccinate, an anticancer drug approved for the treatment of hematological malignancies, has been identified as a potent anti-glioma candidate in various GBM preclinical studies [104,105,146,148]. Omacetaxine mepesuccinate is characterized by a favorable PK profile for CNS penetration [149] and, mechanistically, it targets the ribosomal protein 3 (RPL3), therewith inhibiting protein translation [150]. Other examples of available anticancer agents that have been identified to exhibit potent anti-glioma effect at preclinical level include plicamycin an antineoplastic antibiotic agent [148,151], trametinib a MEK inhibitor [152,153], afatinib an EGFR inhibitor and topotecan a topoisomerase-I inhibitor [105].

A different category of drugs being evaluated concerns non-anticancer agents that may have indirect or off-target effects on glioma cells. In particular, drugs developed for CNS diseases are of interest for glioblastoma due to their optimal PK characteristics for CNS penetration. Their well-established safety profile makes them of interest for combination strategies with anticancer drugs. An example is the recently published study by Bi et al. on the anticancer effects of fluoxetine [154]. Fluoxetine is a second-generation antidepressant drug, which selectively inhibits serotonine reuptake. It possesses a good safety profile and optimal PK characteristics for CNS penetration [155,156]. Preclinical studies in various types of cancer have shown that fluoxetine induces its anticancer effect by inhibiting the NF-κβ signaling and inducing Ca^2+^ related apoptosis [156,157,158]. Furthermore, fluoxetine has been found to act synergistically with temozolomide against glioma cells [154,159]. A retrospective survival analysis revealed that GBM patients taking fluoxetine during their chemo-radiation treatment had a prolonged overall survival [154]. Other non-oncological drugs being investigated as anti-glioma agents include various statins [160], metformin [161,162] and disulfiram [163] as well as drugs developed for treating various pathogens, such as mebendazole, chloroquine and lumefantrine [164,165,166,167]. CNS compounds under investigation include anti-schizophrenia drugs (fluphenazine and fluspirilene) [168] and anti-epileptics (valproic acid [169] and levetericam [170,171]) as well as drugs developed for Alzheimer’s (memantine) [172] or Parkinson’s disease (pimavanserin) [173] (Table 1).

Finally, a clinical trial has been initiated combining nine different non-oncological agents, the CUSP9 treatment protocol, with a low dose of temozolomide in recurrent GBM patients [174]. Adapted versions of the treatment regiments include the CUSP9* [175] and CUSP9v3 [176], with the latter revealing safety of this combination treatment approach. CUSP9v3 is currently being further investigated in a phase 1/2 clinical trial (NCT02770378).

### 4.2. Drug Selection for CNS Delivery

Selecting drug candidates for systemic delivery, which can achieve therapeutic concentrations in brain tumor tissue, may significantly improve the outcomes of clinical studies [177]. This approach requires, already from a preclinical stage, a focus on drugs showing a higher probability of crossing the BBB. Various algorithms and drug discovery tools have been developed to predict the CNS permeability of drugs [178,179,180,181,182,183,184]. Among these is the well-recognized CNS Multiparametric Optimization (CNS-MPO) desirability tool, which is characterized by a simple-to-use design algorithm and multiparameter approach in drug discovery [40]. Wager et al. based this drug discovery method on an algorithm by which molecules can be characterized based on their physicochemical properties to predict CNS penetration [40]. These properties include the partition of coefficient (logP), constant of distribution (logD), polar surface area (PSA), number of hydrogen atoms and acid dissociation constant (pKa) as well as molecular weight (MW) [40]. The algorithm combines these parameters into a single value that provides an estimation of the probability that the drug will reach the CNS. The availability of such scores can aid in selecting compounds of interest in drug repurposing programs for GBM. Additionally, the selection of compounds with negative substrate properties for the drug efflux pumps can help to maintain and prolong therapeutic drug levels in the CNS and contribute to better treatments. Although highly valuable in the pre-selection process, these algorithms and properties do not provide a guarantee for drug CNS penetration. Factors such as the absorption, distribution, metabolism and excretion (ADME) profiles of the drug candidates can strongly influence the ultimate drug concentrations in the tumor tissue. Therefore, it remains essential to perform additional in vivo studies to confirm adequate target delivery and modulation.

### 4.3. The Importance of Phase 0 Clinical Trials in Glioblastoma

Findings derived from pre-clinical drug development studies can be a milestone in bringing promising compounds to clinical implementation [141]. In this process, the PK profile of these compounds has a significant role and confirmation of drug penetration into the tumor tissue at therapeutically effective concentrations provides important support for further development [208]. Various pre-clinical models have been developed to recapitulate the biology of the disease and predict BBB penetration, providing valuable findings for the research community [78,137,182,209,210]. However, these models will not have the weight of evidence of a clinical trial and can only serve as an aid in identifying more or less promising compounds. Currently, a paradigm shift is taking place, where PK/PD and drug delivery studies are forming an essential step in drug development for CNS malignancies. These so-called “window-of-opportunity” studies, or phase 0 trials, are exploratory studies that can promote the rapid clinical implementation of promising preclinical findings on registered compounds and therefore circumvent phase 1 trials given the available knowledge on the safety profiles of these agents [185]. In phase 0 trials, patients are exposed shortly to a drug prior to tumor resection. The resected material is analyzed by liquid chromatography coupled with tandem mass spectrometry (LC-MS/MS) to confirm drug delivery to the tumor, and by additional assays to verify modulation of the intended molecular targets [41]. Upon confirmation of these two key points, eligible patients are subsequently enrolled into a phase 2 study arm or into a different study in order to avoid exposure to an ineffective treatment.

The AZD1775, a first in-class Wee1 inhibitor, is one of the first anticancer agents where the tumor penetration and clinical biological activity were evaluated in recurrent GBM patients [211]. Since then, multiple phase 0/2 clinical trials with registered anticancer agents have been initiated in recurrent glioma patients. Examples include (1) ixazomib, a proteasome inhibitor registered for multiple myeloma and relapsed or refractory systemic light chain (AL) amyloidosis [212,213]; (2) ribociclib, a cyclin-dependent kinase (CDK) 4/6 inhibitor in combination with everolimus an mTOR inhibitor, both registered for advanced or metastatic breast cancer (NCT03834740) [214,215,216]; (3) infigratinib, a kinase inhibitor registered for unresectable locally advanced or metastatic cholangiocarcinoma (NCT04424966) [217]; and (4) pamiparib, a poly(ADP-ribose) polymerase (PARP) 1/2 inhibitor approved in China for the treatment of recurrent advanced ovarian cancer, fallopian tube or primary peritoneal cancer previously treated with two or more lines of chemotherapy (NCT04614909) [218].

These “window-of-opportunity” phase 0 trials, as recently reviewed by Vogelbaum et al. [219], have successfully demonstrated target delivery and drug activity for some of these agents, subsequently allowing them to move to phase 2 clinical investigations. As a next step, even more personalized approaches can be envisioned, in which confirmed drug delivery is a pre-requisite for follow-up treatment with any drug being investigated, therewith accounting for inter-patient variability in BBB functionality. Together, these approaches are highly valuable as phase 0/2 studies cover the unmet need for a rapid early phase drug development track by preventing patient exposure to ineffective treatments, identifying novel drugs for further investigation and thus increasing the success rate of later-stage clinical studies [185].

### 4.4. Clinical Applications of Repurposed Drugs Systemically Delivered in Glioblastoma

As a result of these developments, a plethora of approved drugs, initially developed for other malignancies or non-oncological indications, are now being (re)evaluated as potential treatments for GBM patients [166,194,220,221,222]. An overview of previous and ongoing clinical trials assessing repurposed drugs in GBM patients is presented in Table 2. Such examples include the approved anticancer agent regorafenib, an inhibitor of multiple kinases [223] that is currently being investigated in the phase II/III AGILE trial for newly diagnosed or recurrent GBM (NCT03970447). Previously, a randomized, multicenter, open-label phase 2 trial (REGOMA) indicated enhanced efficacy of regorafenib compared to CCNU in recurrent GBM patients [224]. These findings were further supported by a retrospective clinical study analyzing the clinical outcomes of recurrent GBM patients receiving regorafenib outside clinical trials [225].

Another repurposed registered anticancer drug is abemaciclib [226], which targets the CDK4/6-RB1 signaling pathway and it is under development for brain tumors. Preclinically, abemaciclib was found to reach the brain at therapeutically effective concentrations and exhibit a strong anti-glioma effect [185,227]. The PK and PD profile of abemaciclib was assessed in GBM patients where the drug was detected in the cerebrospinal fluid (CSF) and associated with the inhibition of CDK4/6 [228]. According to Li et al., abemaciclib is considered the most optimal candidate of its class and is currently being investigated as monotherapy as well as in combination with LY3214996 in GBM patients (NCT02981940 and NCT04391595) [185].

Niraparib is a highly selective inhibitor of PARP 1/2 and registered for the treatment of advanced ovarian cancer [229]. In glioblastoma, various PARP inhibitors (PARPi) were clinically tested in patients with recurrent glioblastoma [230]. However, the poor CNS penetration, high affinity to efflux pumps transporters as well as the adverse events caused by the combination of PARPi with temozolomide did not lead to significant therapeutic benefit [231,232,233]. Recently, the implementation of TTF in glioblastoma treatment was associated with the downregulation of breast cancer type 1 susceptibility protein (BRCA1) signaling and the reduction in DNA double-strand break repair capacity [234]. The therapeutic effect of niraparib in combination with TFF is currently being investigated in a phase 2 clinical study (NCT04221503).

Ibrutinib, a small-molecule inhibitor of Bruton’s tyrosine kinase (BTK) and bone marrow X-linked (BMX) non-receptor tyrosine kinase, has been approved for the treatment of patients with chronic lymphocytic leukemia (CLL) and mantle cell lymphoma [235]. In glioblastoma, BMX non-receptor tyrosine kinase is overexpressed, promoting the abnormal activation of the signal transducer and activator of transcription 3 (STAT3), which is involved in self-renewal of glioma stem cells (GSCs) and maintaining GSC tumorigenic potential [236]. Preclinical studies suggest that targeting GSCs through BMX inhibition by ibrutinib may effectively improve GBM treatment [196]. The tolerability and safety of Ibrutinib is currently being investigated in phase 1 clinical study (NCT03535350) in combination with the standard-of-care treatment.

Research interest has also been placed in hydroxyl-chloroquine (CHQ), an anti-malaria drug, of which the anti-tumor effects have been investigated in different types of cancer, including GBM [237,238,239]. CHQ expresses its anticancer effect through the inhibition of autophagy or interference with PI3K/Akt or EGFR signaling pathways in glioma cells [240,241]. A phase 1/2 clinical trial investigated the therapeutic benefit of combining CHQ it to the standard-of-care treatment [242]. Additionally, Compter et al. have shown that, although a slight improvement of the overall survival was observed in GBM patients with EGFRvIII positive tumors, significant side effects may arise by combining CHQ to radiotherapy and TMZ [166].

Hydroxyurea is another FDA-approved compound against various diseases including cancer, which has been found to sensitize glioma cells to temozolomide [243]. A phase 1 clinical study is currently investigating the therapeutic benefit of hydroxyurea in combination with TMZ in recurrent GBM patients (NCT03463733).

Disulfiram (DSF), an approved anti-alcoholism agent, in combination with nutritional copper (Cu) supplement was found to enhance the therapeutic effect of temozolomide in glioma cells [244]. These preclinical data suggest that DSF/Cu in combination with alkylating chemotherapy decreases glioma cell invasion and impairs the DNA repair pathways, thereby improving the efficacy of DNA alkylating agents [163].The safety of this approach was demonstrated in a phase 1 trial for recurrent GBM and the efficacy of this combination was investigated in phase 2 trial, which showed limited activity in the unselected IDH-wild type GBM population [245,246]. Currently, a phase 2 clinical study is ongoing to assess the effect of the DSF/Cu combined with TMZ in GBM patients with unmethylated MGMT promoter (NCT03363659).

A highly interesting approach being investigated in a phase 3 trial is the direct screening of patient-derived tumor cells to determine the most promising anticancer drug for a particular patient (ChemoID) (NCT03632135). This panel of approved anti-cancer agents includes carboplatin, irinotecan, etoposide, carmustine, lomustine, temozolomide, procarbazine, vincristine, imatinib or combinations of these drugs. A personalized pre-screen takes the intertumoral heterogeneity into account and therewith avoids treating patients with drugs for which the tumor is intrinsically resistant. Such approaches are expected to yield much higher response rates [141].

## 5. Novel Approaches for Delivering Repurposed Drugs across the BBB

The limitations in developing novel drugs that overcome the constraints of the BBB brought a new technological era in delivery systems. Novel approaches aim to reinforce the therapeutic potential of drugs lacking favorable PK characteristics for CNS penetration. One such approach is the focused ultrasound sonication (FUS) technology. This non-invasive technology is designed to improve drug delivery to the CNS by opening the BBB with ultrasonic waves [44,247]. Preclinical and clinical studies have shown that FUS technology can enhance the systemic delivery of compounds with poor PK profile, such as paclitaxel, doxorubicin and carboplatin across the BBB into the CNS [52,53,248,249]. The promising results of these preclinical studies has prompted further investigations in multiple clinical studies combining FUS technology to the standard-of-care treatment (NCT04614493) and/or widely used anticancer agents such as carboplatin (NCT04440358) [250] and paclitaxel (NCT04528680) in GBM patients.

Other technologies being developed for improved CNS drug delivery include nanomaterials, such as liposomes and nanoparticles, which can be used as carriers of therapeutic agents [251,252,253]. Encapsulated drugs can be designed to provide higher drug stability together with elevated accumulated concentrations in the tumor tissue and lower drug-related toxicities [254]. The systemic delivery of encapsulated drugs can also be achieved using exosomes [255]. These nano-sized extracellular vesicles can act as nanocarriers releasing therapeutic agents to the tumor cells and improving drug efficacy [256].

Additionally, local delivery methods, such convection-enhanced delivery (CED) as well as intranasal delivery, are being used to circumvent the BBB/BTBB and achieve the direct delivery of repurposed drugs into the (peri)tumoral area. Such examples include topotecan [257], which is delivered via CED against recurrent GBM, as well as perillyl alcohol via intranasal delivery [258]. Similarly, a novel introduced catheter systems for direct delivery of therapeutics to the brain (Neuroinfuse™) aims to improve chronic and acute implantable intra-parenchymal drug delivery [259]. To date, the only FDA-approved local delivery approach against GBM are Gliadel^®^ wafers, showing an enhanced efficacy when combined with standard-of-care treatment [260]. However, this approach has limitations as only patients undergoing a gross total tumor resection are eligible for this treatment and therapeutic benefit is dependent on factors such as age and Karnofsky performance score (KPS) [261].

With the emergence of more and more (repurposed) candidate drugs with potent anti-glioma activity from patient-derived tumor drug screening platforms, and the parallel development of improved CNS delivery techniques, it is expected that the arsenal of compounds becoming available for clinical assessment in GBM patients will greatly expand.

## 6. Conclusions

Glioblastoma has one of the worst prognoses among all cancers. The lack of progress in developing new treatments is inextricably linked to the hurdle of delivering drugs to the CNS as well as the heterogeneity of GBM. Therefore, there remains a high need for therapeutic agents, either as monotherapy or in combination with standard-of-care treatment, from which GBM patients will benefit. Drug repurposing is an important player in this battle, offering novel treatment options with rapid clinical implementation by circumventing the standard drug development process. Key in drug repurposing is the availability of patient-derived, clinically relevant, preclinical model systems that allow for the screening of available drugs and the identification of potent compounds for GBM (subtypes). Moreover, the development of novel algorithms and the available PK data can significantly aid in selecting drug candidates that can effectively cross the BBB. To date, this approach has already led to the identification of candidate drugs registered for various other types of cancer. The validation of drug delivery in relevant in vivo models can further reduce the risk of bringing ineffective drugs to clinical trials. Finally, the clinical implementation of these findings in phase 0/2 trials, as well as the development of novel technologies to improve CNS delivery, is expected to significantly improve the success rate of anti-glioma treatments evaluated in patients.

Taken together, the vast number of registered drugs available for (re)evaluation in clinically relevant GBM model systems, combined with improved tools to predict, validate and achieve CNS penetration, is expected to rapidly increase the entry of more effective compounds against GBM into the clinical arena.

## Figures and Tables

**Table 1:** Examples of oncological and non-oncological repurposed drugs under preclinical investigation against GBM.

Drug Name	Drug Category	Drug Class	Indications	Moa	Moa In Glioma	Reference
Abemaciclib	Oncological	Kinase inhibitors	Breast cancer	CDK4/6 inhibitor	CDK4/6 inhibitor	[185]
Actinomycin-D	Oncological	Antineoplastic antibiotic	Ovarian, testicular, Ewing sarcoma, rhabdomyosarcoma, trophoblastic neoplasms, Wilms tumor	RNA polymerase 1 inhibitor	SOX-2 downregulation	[145]
Afatinib	Oncological	Kinase inhibitors	Non-small-cell lung cancer	EGFR inhibitor	Inhibition EGFRvIII–cMet signaling pathway	[186]
Aprepitant	Non-Oncological	Antiemetics	Nausea/Vomiting	Substance P/NK1 receptor antagonist	NK1 inhibitor	[187]
Auranofin	Non-Oncological	DMARD	Rheumatoid arthritis	Thioredoxin reductase inhibitor	NA	[187]
Captoril	Non-Oncological	ACE inhibitors	Hypertension	ACE inhibitor	ACE, MMPs, AT1 receptors	[187]
Carboplatin	Oncological	Antineoplastic/Platinum analog	Ovarian, lung, head and neck cancer	DNA cross-linking/alkylation	DNA cross-linking/alkylation	[188]
Celecoxib	Non-Oncological	NSAIDs	Osteoarthritis and rheumatoid arthritis	COX-2 inhibitors	COX-1 and -2, carbonic anhydrase-2 and -9	[187]
Chloroquine	Non-Oncological	Antimalarial/ amebicide	Malaria	DNA replicationinhibitor	Inhibition PI3K/Akt or EGFR signaling pathways	[189]
Dabrafenib	Oncological	Kinase inhibitors	Melanoma	BRAF inhibitor	BRAF-inhibitor	[125]
Disulfiram	Non-Oncological	Anti-alcoholism	Alcoholism	ALDH inhibitor	In combination with copper, induces ROS, activates p38 and inhibits NF-κB	[190]
Doxorubicin	Oncological	Anthracyclines	Ovary, prostate, stomach, thyroid, lung, liver; head and neck cancer, multiple myeloma, Hodgkin’s disease, lymphomas, acute lymphocytic leukemia and acute myeloid leukemia	DNA topoisomerase-2 inhibitor	DNA topoisomerase-2 inhibitor	[191]
Etoposide	Oncological	Podophyllotoxin derivatives	Testicular cancer	DNA topoisomerase-2 inhibitor	DNA topoisomerase-2 inhibitor	[192]
Everolimus	Oncological	Kinase inhibitors	Breast cancer	mTOR inhibitor	mTOR inhibitor	[193]
Fluoxetine	Non-Oncological	Antidepressant	Depression	Serotine uptake inhibitor	SMPD1inhibitor	[154]
Fluphenazine	Non-Oncological	Antipsychotic	Schizophrenia	Dopamine D2 receptors inhibitor	Inhibition of mitochondrial CcO and GPCR σ-receptors, increase AMPK activity	[194]
Fluspirilene	Non-Oncological	Antipsychotic	Schizophrenia	Dopamine D2 receptors inhibitor	Inactivation of STAT3	[195]
Ibrutinib	Oncological	Kinase inhibitors	Chronic lymphocytic leukemia and small lymphocytic lymphoma	BTK and BMX inhibitor	BMX inhibitor	[196]
Imatinib	Oncological	Kinase inhibitors	Chronic myeloid leukemia	Bcr-Abl inhibitor	Bcr-Abl and FAK inhibitor	[197]
Infigratinib	Oncological	Kinase inhibitors	Metastatic cholangiocarcinoma	FGFR-1, -2, -3	FGFR-1	[198]
Irinotecan	Oncological	Antineoplastic	Colorectal and pancreatic cancer	Topoisomerase-1 inhibitor	Topoisomerase-1 inhibitor	[199]
Itraconazole	Non-Oncological	Antifungals	Systematic fungal infections	14-α demethylase inhibitor	P-gp efflux transporters, BCRP, hedgehog, 5-lipoxygenase	[187]
Ixazomib	Oncological	Kinase inhibitors	Multiple myeloma	Proteasome subunit beta type-5 inhibitor	Proteasome subunit beta type-5 inhibitor	[200]
Levetericam	Non-Oncological	Anticonvulsants	Epilepsy	Prolong Na+ channel inactivation and GABA transaminase inhibitor	Promoting HDAC4 nuclear translocation and apoptosis	[194,170]
Lumefantrine	Non-Oncological	Antimalarial	Malaria	β-hematin inhibitor	Fli-1 inhibitor	[167]
Mebendazole	Non-Oncological	Anthelmintics	Roundworm and whipworm infections	Microtubules inhibitor	Microtubules inhibitor	[194,201]
Memantine	Non-Oncological	NMDA receptor antagonist	Alzheimer	blocks current flow through channels of NMDA receptors	NA	[172]
Metformin	Non-Oncological	Antidiabetic	Hyperglycemia	Complex 1 of the mitochondrial respiratory chain inhibitor	CLIC-1 mediated ion currentinhibitor	[202]
Minocycline	Non-Oncological	Tetracycline antibiotics	Bacterial infections	Protein synthesis inhibitor	Monocyte, macrophage and microglial inhibition	[187]
Omacetaxine mepessucinate	Oncological	Antineoplastic	Chronic myeloid leukemia	Protein synthesis (RPL3) inhibitor	NA	[104]
Paclitaxel	Oncological	Anti-microtubule agents	Ovarian, breast, and non-small cell lung cancer	Tubulin beta-1 chain inhibitor	Tubulin beta-1 chain inhibitor	[203]
Pimavanserin	Non-Oncological	Atypical antipsychotic	Parkinson	Inverse agonist/antagonist of serotonin 5HT2A and 5HT2C receptors	Ca 2+-calcineurin-NFAT pathway inhibitor	[204,173]
Topotecan	Oncological	Antineoplastic	Ovarian and lung cancer	Topoisomerase 1inhibitor	SUMOylationinhibitor	[205]
Trametinib	Oncological	Kinase inhibitors	Melanoma	MEK inhibitor	MEK inhibitor	[153]
Valproic acid	Non-Oncological	Anticonvulsants	Epilepsy	Histone deacetylase 9 inhibitor	SSADH downregulation	[206]
Vincristine	Oncological	Vinca alkaloids	Acute lymphocytic leukemia, lymphoid blast crisis of chronic myeloid leukemia, and Hodgkin and Non-Hodgkin lymphoma	Tubulin beta chain inhibitor	Tubulin beta chain inhibitor	[207]

CDK4/6: cyclin-dependent kinase 4/6, NK1: neurokinin 1, DMARD: disease modifying anti-rheumatic drug, ACE: angiotensin-converting enzyme, MMPs: matrix metalloproteinases, NSAIDs: non-steroidal anti-inflammatory drugs, COX-2: cyclo-oxygenase-2, mTOR: mammalian target of rapamycin, BTK: Bruton’s tyrosine kinase, BMX: bone marrow tyrosine kinase on chromosome X, FAK: focal adhesion kinase, FGFR: fibroblast growth factor receptors, P-gp: P-glycoprotein, BCRP: breast cancer-resistant protein, RPL3: ribosomal protein 3, EGFR: epidermal growth factor receptor, EGFRvIII: epidermal growth factor receptor variant-III, SUMO: small ubiquitin-like modifier, SMPD1: sphingomyelin phosphodiesterase 1, CLIC-1: chloride intracellular channel 1, ALDH: aldehyde dehydrogenase, ROS: reactive oxygen species, NF-κB: nuclear factor kappa-light-chain-enhancer of activated B cells, ERK1/2: extracellular signal-regulated protein kinases 1 and 2, Bcl-2: B-cell lymphoma 2, Akt: protein kinase B, FOXO3a: forkhead box O3, Fli-1: friendleukemia integration 1, CcO: cytochrome c oxidase, GPCR: G protein-coupled receptors, STAT3: signal transducer and activator of transcription 3, GABA: γ aminobutyric acid, NMDA:N-methyl-D-aspartate, NFAT: nuclear factor of activated T cells, SSRIs: selective serotonin reuptake inhibitors, TCTP: translationally controlled tumor protein, SSADH: succinic semialdehyde dehydrogenase.

**Table 2:** Repurposed drugs under clinical investigation in glioblastoma.

**NCT Code**	**Study Title**	**Interventions**	**Disease**	**Status**	**Clinical Phase**
NCT03834740	A phase 0/2 study of ribociclib (LEE011) in combination with everolimus in preoperative recurrent high-grade glioma patients scheduled for resection	Drug: Ribociclib Drug: Everolimus	Glioblastoma	Recruiting	Phase 0/2
NCT02981940	A phase 0/2 study of abemaciclib in recurrent glioblastoma	Drug: Abemaciclib	Glioblastoma	Recruiting	Phase 0/2
NCT04391595	A phase 0/2 study of LY3214996 (ERK inhibitor) in combination with abemaciclib (CDK4 and 6 inhibitor) in recurrent glioblastoma participants scheduled for resection to evaluate central nervous system (CNS) penetration	Drug: Abemaciclib Drug: LY3214996	Glioblastoma	Recruiting	Early phase 1
NCT04424966	A phase 0 study of infigratinib in recurrent high-grade glioma participants scheduled for resection to evaluate central nervous system (CNS) penetration with PK triggered expansion cohort	Drug: Infigratinib	Glioblastoma	Recruiting	Early Phase 1
NCT04614909	A phase 0/2 clinical trial of pamiparib in newly diagnosed and recurrent glioblastoma patients	Drug: Pamiparib Drug: Olaparib Radiation therapy Drug: TMZ	Glioblastoma	Recruiting	Early Phase 1
NCT01294735	A phase 1 study of MK-4827 in combination with temozolomide in patients with advanced cancer	Drug: Niriparib (MK-4827) Drug: TMZ	Glioblastoma Melanoma	Completed	Phase 1
NCT03535350	Ibrutinib with radiation and temozolomide in patients with newly diagnosed glioblastoma	Drug: Ibrutinib Radiation Drug: TMZ	Glioblastoma	Recruiting	Phase 1
NCT03463733	Hydroxy-urea and temozolomide in patients with a recurrent malignant brain tumor (glioblastoma) (HUTMZ)	Drug: Hydroxyurea Drug: TMZ	Glioma Glioblastoma	Recruiting	Phase 1
NCT02770378	A proof-of-concept clinical trial assessing the safety of the coordinated undermining of survival paths by 9 repurposed drugs combined with metronomic temozolomide (CUSP9v3 treatment protocol) for recurrent glioblastoma	Drug: TMZ Drug: Aprepitant Drug: Minocycline Drug: Disulfiram Drug: Celecoxib Drug: Sertraline Drug: Captopril Drug: Itraconazole Drug: Ritonavir Drug: Auranofin	Glioblastoma	Completed	Phase 1/2
NCT04440358	Assessment of safety and feasibility of Exablate blood–brain barrier disruption (BBBD) with microbubbles for the treatment of recurrent glioblastoma (rGBM) in subjects undergoing carboplatin monotherapy	Device: Exablate BBBD Drug: Carboplatin	Glioblastoma	Recruiting	Phase 1/2
NCT04528680	Phase 1/2 trial of blood–brain barrier opening with an implantable ultrasound device SonoCloud-9 and treatment with albumin-bound paclitaxel in patients with recurrent glioblastoma	Device: Sonication for the opening of blood–brain barrier Drug: albumin-bound paclitaxel	Glioblastoma	Recruiting	Phase 1/2
NCT04051606	Regorafenib in bevacizumab refractory recurrent glioblastoma	Drug: Regorafenib	Recurrent Glioblastoma	Recruiting	Phase 2
NCT03970447	A trial to evaluate multiple regimens in newly diagnosed and recurrent glioblastoma (GBM AGILE)	Drug: TMZ Drug: Lomustine Drug: Regorafenib Radiation	Glioblastoma	Recruiting	Phase 2/3
NCT02926222	Regorafenib in relapsed glioblastoma (REGOMA)	Drug: Regorafenib Drug: Lomustine	Glioblastoma	Active, non-recruiting	Phase 2
NCT04221503	A phase 2 study evaluating the efficacy and safety of niraparib and tumor-treating fields in recurrent glioblastoma	Drug: Niraparib Device: Optune	Glioblastoma Recurrent Glioblastoma	Recruiting	Phase 2
NCT03243851	Study on low-dose temozolomide plus metformin or placebo in patient with recurrent or refractory glioblastoma (METT)	Drug: TMZ +Metformin Drug: TMZ +Placebo	Glioblastoma	Recruiting	Phase 2
NCT03363659	Disulfiram and copper gluconate with temozolomide in unmethylated glioblastoma multiforme	Drug: Disulfiram Dietary Supplement: Copper gluconate Drug: TMZ	Glioblastoma	Recruiting	Phase 2
NCT02432417	The addition of chloroquine to chemoradiation for glioblastoma	Drug: Chloroquine	Astrocytoma, Grade IV	Not yet recruiting	Phase 2
NCT03632135	Standard chemotherapy vs. chemotherapy guided by cancer stem cell test in recurrent glioblastoma (CSCRGBM)	Diagnostic Test: ChemoID assay Drug: Chemotherapy	Recurrent Glioblastoma	Active, non-recruiting	Phase 3
